# A cost-benefit/cost-effectiveness analysis of proposed supervised injection facilities in Montreal, Canada

**DOI:** 10.1186/1747-597X-8-25

**Published:** 2013-07-09

**Authors:** Ehsan Jozaghi, Andrew A Reid, Martin A Andresen

**Affiliations:** 1 School of Criminology, Simon Fraser University, 8888 University Drive, Burnaby, British Columbia, Canada V5A 1S6

**Keywords:** Supervised injection facility, Harm reduction, Cost-benefit, Cost-effectiveness

## Abstract

**Background:**

This paper will determine whether expanding Insite (North America’s first and only supervised injection facility) to more locations in Canada such as Montreal, cost less than the health care consequences of not having such expanded programs for injection drug users.

**Methods:**

By analyzing secondary data gathered in 2012, this paper relies on mathematical models to estimate the number of new HIV and Hepatitis C (HCV) infections prevented as a result of additional SIF locations in Montreal.

**Results:**

With very conservative estimates, it is predicted that the addition of each supervised injection facility (up-to a maximum of three) in Montreal will on average prevent 11 cases of HIV and 65 cases of HCV each year. As a result, there is a net cost saving of CDN$0.686 million (HIV) and CDN$0.8 million (HCV) for each additional supervised injection site each year. This translates into a net average benefit-cost ratio of 1.21: 1 for both HIV and HCV.

**Conclusions:**

Funding supervised injection facilities in Montreal appears to be an efficient and effective use of financial resources in the public health domain.

## Introduction

The spread of infectious diseases among injection drug users (IDUs) is a serious public health concern. In a recent systematic review and meta-analysis analyzing research studies from Western Europe, Asia, Latin America, Australasia, Eastern Europe, and North America it was found that acquired immunodeficiency syndrome (AIDS) and a number of other blood-borne diseases transmitted through shared needles and syringes are of the leading causes of death across 67 cohorts of IDUs [1]. The extent of these health concerns is particularly troubling. The World Health Organization notes that globally approximately 16 million people inject drugs and of those 3 million are living with the human immunodeficiency virus (HIV) [2]. While rates of injection drug use and the contraction of infectious diseases may be elevated in certain regions of the world, first world countries are not exempt from these problems.

In Canada, for example, based on the most recent data available there were 1695 deaths attributable to illegal drug use in 2002 [3]. AIDS is a significant cause of death related to illicit drugs, with 87 identified AIDS deaths attributed to injection drug use in that year [3]. Further, approximately 70 percent of new hepatitis C virus (HCV) infections in Canada are attributable to injection drug use and needle sharing [4]. The spread of infectious diseases is not, however, the only concern.

These problems are of particular concern in Canada’s urban areas where IDU populations are large. In Montreal, Quebec for example, the IDU population has been estimated at between 4,300 and 12,500 individuals [5]. This elevated IDU population translates into increased infection rates. In fact, HIV and HCV prevalence rates for IDUs in Montreal have been estimated to be as high as 18 percent and 68 percent, respectively [6]. Despite having various harm reduction strategies implemented in the greater Montreal area (such as 11 needle depots, 11 community health centres, two methadone clinics and 150 pharmacies that distribute approximately 800,000 syringes), IDUs continue to share their injection drug equipment [7,8]. Recent data from Montreal indicates rising incidents of HIV and HCV among IDUs from 2004 to 2006 [9,10].

Recognizing these growing concerns, the Quebec government has shown interest in adopting further strategies to reduce the spread of infectious diseases in its IDU population. One such strategy is a supervised injection facility such as Insite that has been in operation in Vancouver, British Columbia since 2003—North America’s first and only legal supervised injection facility. Though its continued operation has faced legal obstacles, in a recent Supreme Court of Canada case the continuation of Insite’s operation was upheld (PHS Community Services v. Attorney General of Canada, BCSC, 2008; Canada [Attorney General] vs PHS Community Services Society, 2011 SCC 44). Buoyed by this most recent Supreme Court of Canada ruling, and in order to reduce the public health and fiscal impacts of injection drug use, the Quebec government has shown interest in opening supervised injection facilities (SIFs) in Montreal [11]. According to Quebec’s Health and Social Services Minister, Yves Bolduc, SIFs “will offer services that will monitor the health of drug addicts and encourage them to seek detoxification and rehabilitation” [10]. Health officials in the City of Montreal have already proposed three SIFs plus a mobile SIF that will attempt to reach the IDU population [10]. At this point, however, it is unclear exactly what magnitude of impact the proposed SIFs may have for the City of Montreal. An obvious question that is of great concern to City officials is: Are SIFs an efficient and effective use of financial resources? This issue is of critical importance because of the recently established more restrictive rules made by the Federal government in order to establish a supervised injection facility [12,13].

The current paper presents cost-effectiveness and cost-benefit analyses for various SIF operation scenarios in Montreal in an effort to inform public policy on this complex issue. Specifically, the analyses estimate the number of new HIV and HCV infections prevented as a result of operating SIFs using mathematical modelling with conservative parameter estimates. The dollar costs of illnesses avoided are compared to the operational cost of a SIF. The analyses are then extended to consider the impact of opening additional SIFs. As long as the marginal benefits outweigh the marginal costs of additional SIFs hours and locations, the expansion of SIF should be recommended. At some point, however, it is expected that the marginal cost of expansion will exceed the marginal benefits.

### Related studies

Due to inherent issues associated with the legality of injection drug use in many countries around the world, few SIFs have been put into practice [14]. Consequently, research examining the cost-benefit and cost-effectiveness characteristics of SIFs is very limited [13]. The first published economic analysis of the SIF portion of Insite considered a complex dynamic compartmental simulation model for the City of Vancouver as a whole and projected new HIV and hepatitis C infections over a 10-year time horizon with and without Insite [15]. In their baseline model, the authors estimated 1191 new HIV and 54 new hepatitis C cases were averted, a simple average over the 10 years of 120 new cases of HIV each year. Considering the lifetime cost of a new HIV infection at $210 555 [16], these averted cases led to an annual savings of $25 million, and a benefit-cost ratio of 16.84, assuming a $1.5 million cost of operations for the SIF portion of Insite [17].

Therefore, this model predicted that Insite prevented 80 percent of all new IDU cases of HIV each year for all of British Columbia when approximately 5 percent of all injections in Vancouver’s DTES took place within Insite. This model is complex, dynamic, and considers a score of variables, but the results are quite simply not believable.

Another economic costing study showed that Insite as a whole (SIF, needle exchange program, etc.) was extremely cost-effective, but the SIF alone contributed very little: 83.5 new HIV cases were averted each year, with 2.8 attributed to the SIF [16]. Considering the values used above, this led to a benefit-cost ratio of 0.37. Clearly, these latter results indicate that Insite should not continue its SIF activities if costs are to determine that choice.

Using a mathematical modeling approach, a cost-benefit and cost-effectiveness analysis of Insite was also undertaken that resulted in much more believable results [17]. These researchers considered 4 mathematical models used in the needle exchange evaluation literature, varying parameter values in a sensitivity analysis, and found that 19 to 57 new cases of HIV are averted, an average of 35 cases averted each year. This led to benefit-cost ratios ranging from 1.94 to 5.8, an average of 3.56.

Most recently, researchers conducted a costing study to determine if an expansion of the SIF program could improve upon the cost-benefit and cost-effectiveness estimates found in the earlier research [13]. The study also explored the degree to which the services could be expanded before diminishing returns would take effect. The analyses demonstrated that while expanding Insite may lead to further cost savings, this result was dependant on behavioral changes in the IDU population [13]. In other words, Insite would have to recruit new users from the IDU population in order for additional cost-savings to be achieved.

An obvious question emerges at this point in the discussion: why do one set of results [16] differ so much from the other studies [13,15,17]? The answer is simple. The research that did not support the SIF portion of Insite [16] did not consider behavioral changes of IDUs with regard to needle-sharing in his models. Overall, these studies indicated that Insite was cost-saving for society, but for different reasons: the SIF [13,15,17] and the needle exchange program [16]. Because the SIF within Insite is the contentious aspect of Insite, we focus our analysis on the SIF. We consider this reasonable because these two functions of Insite are separable and distinguishable. And because of the empirical support for changes in the behaviour of IDUs [17] and the realistic nature of the simple mathematical modeling approach [13,17], we follow the methodology of these researchers.

While most of the studies related to Insite found positive cost-benefit and cost-effective results when considering the Vancouver-based SIF, it would be irresponsible to assume that the same benefits would be experienced for the implementation of SIFs in other geographic locations. As a result, with increasing interest in adopting SIFs in the Montreal area, cost-benefit and cost-effectiveness analyses in that specific location are warranted.

## Methods

### Model

For the purposes of the current analysis, it was necessary to rely on a model that could reflect the effects of providing clean injection equipment and adopting safer injecting behaviors within its scope of calculation. Drawing from the methodological approach adopted in recent research on the economic impact of a needle exchange program in Edmonton, Alberta, Canada [17], the current study uses a mathematical model to estimate the number of HIV and HCV infections that could be prevented through the establishment of a SIF in Montreal [18]. The number of new HIV infections avoided, (*A*), is calculated as follows:

$$A=\mathrm{INsd}\left[1-{\left(1-\mathrm{qt}\right)}^{m}\right],$$

where (*I*) is the IDU population that is HIV or HCV negative, (*N*) is the number of needles in circulation, (*s*) is the needle sharing rate, (*d*) is the percentage of needles not cleaned before use, (*q*) is the HIV or HCV prevalence in the IDU population, (*t*) is the probability of HIV or HCV transmission when using an HIV/HCV infected needles, and (*m*) is the number of sharing partners when injections are shared.

### Variables and parameters

The values of the variables and parameters identified in the above equation were derived from Montreal-specific estimates, including published data; however, when city-specific data were not available, values from the medical and scientific literature were used as estimates (see Table 1).

**Table 1:** Sources for variables used in mathematical modeling

**Variable**	**Value**	**Source**
Proportion of IDUs HIV- (I)	81.20%	Généreux et al. [19]; Broadhead, Kerr, Grund & Altice [20]
Rate of Needle sharing (s)	35%	Bruneau et al. [9]; De et al. [8]; Généreux et al. [19]
Number of needles in circulation (N)	800000	Morissette et al. [7]
Percentage of needles not cleaned (d)	17.00%	Jacobs et al. [18]; Kaplan and O'Keefe [21]
Probability of HIV infections from a single injection (t)	0.67%	Kaplan and O'Keefe [21]
Number of sharing partners (m)	1.38	Jacobs et al. [18]
Proportion of IDUs HIV+ (q)	18.80%	Broadhead et al. [20]; Généreux et al. [19]
Proportion IDUs HCV- (I)	30.00%	De et al. [8]; Broadhead et al. [20]; Généreux et al. [19]
Proportion of IDUs HCV+ (q)	70.00%	De et al. [8]; Broadhead et al. [20]; Généreux et al. [19]
Probability of HCV infection from single injection (t)	3%	Gore & Bird [22]

When several estimates were available in the medical and scientific literature, selection preference was given to values leading to lower bound benefits, making the estimates conservative. At its core, the cost-benefit and cost-effectiveness analysis of this study relied on the number of HIV and HCV infections prevented. In addition, however, the lifetime costs of treating HIV and HCV infections, the operational costs of the potential SIFs in Montreal, new HIV and HCV cases prevented for each additional facility, and finally, the desirable number of facilities were all required for complete model specification.

#### Infectious disease cases prevented

With respect to previous costing studies of SIFs, it has been demonstrated that it is safe to presume that SIFs are able to prevent the risk of new HIV cases because there will be a certain number of known “clean” injections (not shared) as opposed to “dirty” injections (shared) outside of the facility [13,16,17]. In line with previous literature then, the current study employed a point estimate of 0.3 for behavioral change [13,15,16]. To provide a more conservative estimate, an odds ratio of 0.3 was only used for the first and second potential SIFs in Montreal; this was done to limit the number of new users of the Montreal SIFs simply because we cannot expect all IDUs to start using such facilities.

Using the same odds-ration as the Vancouver study that estimated 20–30 new HIV infections prevented, the mathematical model used in the current analysis estimated that the number of new HIV infections would fall from 67 to 53 with the establishment of the first SIF [23]. This represented a reduction of 14 new HIV cases per year using the model [18]. In addition, the model used [18] estimated that 84 new cases of HCV would be averted with the establishment of the first SIF in Montreal. Considering the parameter values and the model employed here, the predicted decline in HCV is from 410 to 326. As with the study of Vancouver’s SIF, we do not consider the impact of the SIF on secondary transmissions, such as those through sexual contact, because reliable data are not available.

#### The medical cost of New HIV and HCV cases

The range of lifetime cost-savings (in 2012 dollars) from averted cases of HIV is great, ranging from $174,410 [24], to US$200,000 [25-27], to more than $289,970 when considering the very successful HAART program [28]. However, the current study assumed a lower cost-savings for HIV infections among IDUs (recognizing that IDUs may experience certain self-imposed barriers or other societal limitations making it less likely for an IDU to take full advantage of the medical system), a lower bound value of $210, 555 was chosen [29]. This value is based on the most recent research in this area [13,16,30].

With respect to costing studies for HCV, the cost-savings range from $20,000 per completed patient course of treatment [31], to $30,000 [28], to more than $69,188 [32]. Once again, here a conservative figure of $35,143 (2012 Dollars), as reported in [33], was chosen.

#### New infectious disease cases

Because no other study has calculated the number of sharing partners in an IDU population, the current model drew from [18] to obtain a value of 1.38. Further, although (*d*)—the percentage of needles not cleaned before use—was 50 percent in [18], the current analysis used the more conservative estimate of 17 percent that was implemented in [21] to make our estimates that much more conservative. The number of total injections within Montreal was also unavailable; therefore, the current study used the product of the number of IDUs (4,300) and the number of injections per year (913) to arrive at a parameter estimate of 3.926 million injections [18,34-36].

#### Cost of SIFs

In order to estimate the cost of establishing SIFs, the analysis must consider the variable and fixed operating cost of existing SIFs. As a result, the study drew these parameters from Insite, the only available comparison facility. The total annual operating cost of Insite in Vancouver is estimated to be $3 million [13,16,37]. This $3 million figure includes primary healthcare, peer counselling, education, housing services, public health screening (immunisation and diagnostic), addiction counselling and case management [13,16,37].

The annual operating cost of Insite in terms of property rental, and supervised injection facility provisions such as injection kits (e.g., insulin syringes with attached needles, bottles of sterile water for injection, latex condoms, alcohol swaps, cost of disposal of used syringes), staff salaries, and equipment purchases is estimated to be $1.53 million [13]. When considering the expansion of Insite from 18 to 24 hours, the operational cost of Insite reaches $2.182 million (2012 dollars)–a one-third increase in the hours of operation [13]. As a result, the current study used $2.182 million for each (proposed) SIF implemented in Montreal. For simplicity, we assume that the Montreal SIFs would provide the same injection kits and have similar costs associated with property rental, staff salaries, and equipment purchases overall—the same service provision as in the Vancouver SIF. This is believed to be a conservative estimate because, according to the Canada Mortgage & Housing Corporation report, rental vacancy rates are higher in Montreal, resulting in lower commercial rent than Vancouver [38]. Further, British Columbia has the highest paid registered nurses and staff in the country [39].

## Results and discussion

The model used here [18], predicted the number of new HIV and HCV cases prevented based on the needle sharing rate. This included the impact of behavioral changes in injection activities outside of the SIF. The behavioral change, according to Table 2 and Table 3, was only considered twice (once for the first SIF and later for the second SIF)—this modeling decision is apparent in the marginal number of new HIV cases averted in Tables 3, 4 and 5. This calculation of behavioral impact is based on a conservative odds-ratio that falls within the limit specified by Kerr et al. (2005) [40].

**Table 2:** **The cumulative annual cost saving, cost - effectiveness and cost – benefit of SIF in Montreal using the Jacobs et al.**[18]**model**

**Variables**	**Annual cost of operation**	**Sharing rate**	**# of HIV averted**	**# of HCV averted**	**HIV cost saved**	**HCV cost saved**	**Cost-effectiveness ratio HCV**	**Cost-effectiveness ratio HIV**	**Cost-benefit ratio HCV**	**Cost-benefit ratio HIV**
Post SIF	$2,182,800	35%	14	84	$764,970	$769,212	$25,986	$155,914	1.35	1.35
Two SIF	$4,365,600	28%	26	162	$1,108,830	$1,327,566	$26,948	$167,908	1.25	1.25
Three SIF	$6,548,400	21%	32	195	$189,360	$304,485	$33,582	$204,637	1.03	1.03
Four SIF	$8,731,200	18%	37	227	-$940,665	-$753,739	$38,463	$235,978	0.89	0.89
Five SIF	$10,914,000	16%	43	261	-$1,860,135	-$1,741,677	$41,816	$253,814	0.83	0.83
Six SIF	$13,096,800	13%	48	294	-$2,990,160	-$2,764,758	$44,547	$272,850	0.77	0.77
Seven SIF	$15,279,600	10%	53	327	-$4,120,185	-$3,787,839	$46,727	$288,294	0.73	0.73
Average	$8,731,200	7%	36	221	-$1,151,220	-$964,597	$39,508	$242,533	0.87	0.87

**Table 3:** **The marginal annual cost saving, cost - effectiveness and cost – benefit of SIF in Montreal using the Jacobs et al.**[18]**model**

**Variables**	**Annual cost of operation**	**Sharing rate**	**Marginal # of HIV averted**	**Marginal # of HCV averted**	**Marginal**	**Marginal**	**Marginal**	**Marginal**	**Marginal**	**Marginal**
					**HIV cost saved**	**HCV cost saved**	**cost-effectiveness ratio HCV**	**cost-effectiveness ratio HIV**	**cost-benefit ratio HCV**	**cost-benefit ratio HIV**
Post SIF	$2,182,800	35%	14	84	$764,970	$769,212	$25,986	$155,914	1.35	1.35
Two SIF	$2,182,800	28%	12	78	$343,860	$558,354	$27,985	$181,900	1.26	1.16
Three SIF	$2,182,800	21%	6	33	-$919,470	-$1,023,081	$66,145	$363,800	0.53	0.57
Four SIF	$2,182,800	18%	5	32	-$1,130,025	-$1,058,224	$38,463	$436,560	0.52	0.48
Five SIF	$2,182,800	16%	6	34	-$1,200,540	-$987,938	$41,816	$363,800	0.55	0.58
Six SIF	$2,182,800	13%	5	33	-$1,130,025	-$1,023,081	$66,145	$436,560	0.53	0.57
Seven SIF	$2,182,800	10%	5	33	-$919,470	-$1,023,081	$66,145	$436,560	0.53	0.57
Average	$2,182,800	7%	8	47	-$498,360	-$531,079	$46,442	$272,850	0.76	0.77

**Table 4:** The sensitivity analysis at 45% sharing rate for marginal annual cost saving, cost - effectiveness and cost – benefit of SIF in Montreal

**Variables**	**Annual cost of operation**	**Sharing rate**	**# of HIV averted**	**# of HCV averted**	**HIV cost saved**	**HCV cost saved**	**Cost-effectiveness ratio HCV**	**Cost-effectiveness ratio HIV**	**Cost-benefit ratio HCV**	**Cost-benefit ratio HIV**
Post SIF	$2,182,800	45%	19	115	$1,817,745	$1,858,645	$18,980	$114,884	1.85	1.8
Two SIF	$2,182,800	35%	16	99	$1,186,080	$1,296,357	$22,048	$136,425	1.6	1.5
Three SIF	$2,182,800	27%	7	43	-$708,915	-$671,651	$50,763	$311,829	0.7	0.68
Four SIF	$2,182,800	23%	7	43	-$708,915	-$671,651	$50,763	$311,829	0.7	0.68
Five SIF	$2,182,800	20%	7	43	-$708,915	-$671,651	$50,763	$311,829	0.7	0.68
Six SIF	$2,182,800	16.00%	7	43	-$708,915	-$671,651	$50,763	$311,829	0.7	0.68
Seven SIF	$2,182,800	12%	7	43	-$708,915	-$671,651	$50,763	$311,829	0.7	0.68
Average	$2,182,800	9%	10	61	-$540,750	-$203,253	$42,120	$258,636	1	0.95

**Table 5:** The sensitivity analysis at 25% sharing rate for marginal annual cost saving, cost - effectiveness and cost – benefit of SIF in Montreal

**Variables**	**Annual cost of operation**	**Sharing rate**	**# of HIV averted**	**# of HCV averted**	**HIV cost saved**	**HCV cost saved**	**Cost-effectiveness ratio HCV**	**Cost-effectiveness ratio HIV**	**Cost-benefit ratio HCV**	**Cost-benefit ratio HIV**
Post SIF	$2,182,800	25%	10	64	-$77,250	$66,352	$34,106	$218,280	1.03	1
Two SIF	$2,182,800	20%	9	55	-$287,805	-$249,935	$39,687	$242,533	0.89	0.87
Three SIF	$2,182,800	15%	4	24	-$1,340,580	-$1,339,368	$90,950	$545,700	0.39	0.39
Four SIF	$2,182,800	13%	4	24	-$1,340,580	-$1,339,368	$90,950	$545,700	0.39	0.39
Five SIF	$2,182,800	11%	4	24	-$1,340,580	-$1,339,368	$90,950	$545,700	0.39	0.39
Six SIF	$2,182,800	9.00%	4	24	-$1,340,580	-$1,339,368	$90,950	$545,700	0.39	0.39
Seven SIF	$2,182,800	7%	4	24	-$1,340,580	-$1,339,368	$90,950	$545,700	0.39	0.39
Average	$2,182,800	14%	6	34	-$919,470	-$987,938	$64,200	$363,800	0.55	0.58

As expected, the results presented in Table 2 and Table 3 show that increasing the scope of SIFs through site expansion would result in a decrease of HIV infection cases. The model predicts: 14–53 fewer HIV cases and 84–327 fewer HCV cases annually, with the marginal range being much smaller: 5–14 fewer HIV cases and 33–84 fewer HCV cases annually.

This range disparity, as outlined in Table 2 and Table 3, translates into substantial differences between the economic evaluation of SIFs with respect to the cumulative versus marginal estimates: the total effect of establishing SIFs and the effect of establishing each subsequent SIF, respectively.

For example, according to Table 3, the cumulative annual estimates of new HIV cases averted, translates into a cost savings for society ranging from $0.764 million (benefit) for the first SIF to -$4.1 million (loss) for the seventh SIF. Benefit-cost ratios range from 1.35 to 0.73, and cost-effectiveness values range from $155,914 to $288,294 (cost per lifetime treatment). The cumulative annual estimates of new HCV cases averted translate into a cumulative cost savings that range from $0.769 million (benefit) for the first SIF to -$3.7 million (loss) for the seventh SIF. Benefit-cost ratios range from 1.35 to 0.73, and incremental cost-effectiveness values range from $25,986 to $46,727 (cost per lifetime treatment).

In contrast, the marginal estimates of Montreal’s SIF expansion translate into a much smaller return. This is particularly true with respect to its benefit-cost and cost-effectiveness ratios. For instance, the marginal benefit-cost ratio varies from 1.35 to 0.77 for HIV and 1.35 to 0.76 for HCV. The marginal cost-effectiveness value for HIV ranges from $155,914 to $436,560 (cost per life- time treatment). The HCV marginal cost-effectiveness value ranges from $25,986 to $66,145 (cost per lifetime treatment).

Furthermore, Table 2 and Table 3 show that both cumulative benefit-cost ratios dwindle after the third SIF. For example, Table 2 shows that a cost savings of $189,360 is present for the third SIF (HIV) results, but the further expansion to four SIFs leads to a $0.940 million loss. A similar loss due to SIF expansion can be seen for HCV where a $304,485 cost saving (for the third SIF) changes to a 0.753 million dollar loss (for the fourth SIF). More specifically, the benefit-cost ratio for both HIV and HCV diminish after the third SIF (from 1.03 to 0.89). Incremental cost-effectiveness ratios also diminish after the third SIF with HIV ($204,637 to $235,978 cost per lifetime treatment) and HCV ($33,582 to $38,463 cost per life- time treatment). This means that they both exceed their cost-effectiveness ratios of $210, 555 and $35,143 respectively.

Finally, sensitivity analysis was conducted for the models employed. These employed different initial needle-sharing rates (see Tables 4, 5, 6 and 7). Similar to [17] and [13], the current analysis used 20 and 40 per cent initial needle-sharing rates. Convincingly, the results from both the baseline and sensitivity analysis in these analyses demonstrate that the establishment of an SIF program in Montreal would save tax payers money.

**Table 6:** The sensitivity analysis at 45% sharing rate for cumulative annual cost saving, cost - effectiveness and cost – benefit of SIF in Montreal

**Variables**	**Annual cost of operation**	**Sharing rate**	**# of HIV averted**	**# of HCV averted**	**HIV cost saved**	**HCV cost saved**	**Cost-effectiveness ratio HCV**	**Cost-effectiveness ratio HIV**	**Cost-benefit ratio HCV**	**Cost-benefit ratio HIV**
Post SIF	$2,182,800	45%	19	115	$1,817,745	$1,858,645	$18,980	$114,884	1.85	1.8
Two SIF	$4,365,600	35%	35	214	$3,003,825	$3,155,002	$20,400	$124,731	1.7	1.7
Three SIF	$6,548,400	27%	42	256	$2,294,910	$2,448,208	$25,580	$155,914	1.4	1.4
Four SIF	$8,731,200	23%	49	299	$1,585,995	$1,776,557	$29,201	$178,188	1.2	1.2
Five SIF	$10,914,000	20%	56	341	$877,080	$1,069,763	$32,006	$194,893	1.1	1.1
Six SIF	$13,096,800	16.00%	63	384	$168,165	$398,112	$34,106	$207,886	1	1
Seven SIF	$15,279,600	12%	70	427	-$540,750	-$273,539	$35,784	$218,280	0.96	0.98
Average	$8,731,200	9%	48	291	$1,375,440	$1,495,413	$30,004	$181,900	1.16	1.17

**Table 7:** The sensitivity analysis at 25% sharing rate for cumulative annual cost saving, cost - effectiveness and cost – benefit of SIF in Montreal

**Variables**	**Annual cost of operation**	**Sharing rate**	**# of HIV averted**	**# of HCV averted**	**HIV cost saved**	**HCV cost saved**	**Cost-effectiveness ratio HCV**	**Cost-effectiveness ratio HIV**	**Cost-benefit ratio HCV**	**Cost-benefit ratio HIV**
Post SIF	$2,182,800	25%	10	64	-$77,250	$66,352	$34,106	$218,280	1.03	1
Two SIF	$4,365,600	20%	19	119	-$365,055	-$183,583	$36,686	$229,768	0.96	0.92
Three SIF	$6,548,400	15%	23	142	-$1,705,635	-$1,558,094	$46,156	$284,713	0.76	0.74
Four SIF	$8,731,200	13%	27	166	-$3,046,215	-$2,897,462	$52,598	$323,377	0.67	0.65
Five SIF	$10,914,000	11%	31	190	-$4,386,795	-$4,236,830	$57,442	$352,065	0.61	0.6
Six SIF	$13,096,800	9%	35	213	-$5,727,375	-$5,611,341	$61,487	$374,194	0.57	0.56
Seven SIF	$15,279,600	7%	39	237	-$7,067,955	-$6,950,709	$71,735	$436,560	0.55	0.54
Average	$8,731,200	14%	26	154	-$3,256,770	-$3,319,178	$56,696	$335,815	0.62	0.63

The estimates of 130–140 HIV reductions and 840 HCV reductions over 10 years (with consideration for growth in IDUs population) is still cost effective for the first three SIFs. Results ranged from $155,914 to $204,638 for HIV (cost per lifetime treatment) and $33,582 to $33,582 for HCV (cost per lifetime treatment). Further, the benefit-cost ratio is above 1 for both HIV and HCV for the first three SIF over 10 years of establishment (see Table 8).

**Table 8:** **The cumulative ten years cost - effectiveness and cost – benefit of sif in montreal using the Jacobs et al.**[18]**model**

**Variables**	**Annual cost of operation**	**# of HIV averted**	**# of HCV averted**	**Cost-effectiveness ratio HCV**	**Cost-effectiveness ratio HIV**	**Cost-benefit ratio HCV**	**Cost-benefit ratio HIV**
Post SIF	$21,828,000	140	840	$25,986	$155,914	1.35	1.35
Two SIF	$43,656,000	260	1620	$26,948	$167,908	1.25	1.25
Three SIF	$65,484,000	320	1950	$33,582	$204,637	1.03	1.03
Four SIF	$87,312,000	370	2270	$38,463	$235,978	0.89	0.89
Five SIF	$109,140,000	430	2610	$41,816	$253,814	0.83	0.83
Six SIF	$130,968,000	480	2940	$44,547	$272,850	0.77	0.77
Seven SIF	$152,796,000	530	3270	$46,727	$288,294	0.73	0.73
Average	$87,312,000	360	2210	$39,508	$242,533	0.87	0.87

## Conclusions

The current analysis set out to assess whether establishing a SIF in Montreal would have a (net) positive fiscal impact on society. In particular, it assessed whether or not this policy initiative would save public health care funds by averting new HIV and HCV infections. Moreover, upon completion of the economic evaluation for the proposed SIF in Montreal, the optimal number of SIFs was assessed based on marginal cost-saving, cost-effectiveness, and benefit-to-cost ratios.

The results presented here suggest that establishing SIFs in Montreal will benefit the publically funded health care system. Further, the implementation of additional SIFs would serve a as a fiscally responsible course of action. It should be noted that although expansion beyond the third SIF location may not provide the same economic return, it may still be considered cost-effective (even if it is not cost saving) given that the analyses used highly conservative estimates in the baseline calculations. For example, the potential for cost-savings with respect to cellulitis, subcutaneous abscesses, endocarditis, and incidence of soft-tissue infections averted were not considered in the calculations.

In addition, for incidents of HIV and HCV, the lower bound estimates were considered. For example, when considering HIV, the current analyses omitted the parameters found to be specified in the HAART program and instead, relied on the lower cost estimates for HIV and HCV. Hence, only at very high levels of coverage is there a diminishing return in the number of new HIV and HCV infections averted for each dollar invested. Moreover, even if the calculations predicted that the expansion beyond the fourth SIF may not be economically desirable (because the benefit-to-cost ratio or cost-effectiveness ratios are not favourable), they could become so in the future because other studies have shown that future benefits will outweigh the start-up costs [41]. As a result, the current model most certainly underestimated the benefits.

While some researchers remain sceptical with respect to the economic rationale for ancillary SIFs [16,30], it should be noted that other models used in recent research have consistently demonstrated the benefits of such expansion [13]. The main difference between the current study’s findings and those in [16,30] is the consideration of behavioral change. Behavioral change has been shown to be an important aspect of the result not only in this costing study, but other studies as well [13,17]. The conditions under which the establishment of a SIF will be economically efficient and cost-saving stem from two sources; one being the provision of clean injecting equipment, and the other being the easier accessibility of services that translate into transformative changes in IDUs behavior (e.g., injection behavior outside Insite becomes less risky).

In sum, establishing SIFs in Montreal has been shown to be cost-saving and the results presented here fall within the range of existing cost-effectiveness/cost-benefit studies despite the variation in methodologies employed. Specifically, this paper has shown that the number of new HIV and HCV infections averted, and the associated cost-savings, are more than enough to cover the cost of operating more than one SIF in Montreal. Therefore, if one accepts the scientific evidence behind the behavioral change in the IDU population, there is a substantial case for establishing a SIF in Montreal. This should serve as an encouraging result for policy makers who seek to find practical, cost-effective solutions to serious health care problems in a climate of scarce public resources.

## Competing interests

The authors declare that they have no competing interests.

## Authors’ contributions

EJ: data gathering, performed the statistical analysis; AAR: drafted the manuscript; MAA: methodological study design and drafting of manuscript. All authors read and approved the final manuscript.
